# Chestnut (*Castanea sativa* Mill.) leaf extract and pure castalagin reduce bacterial colonisation and the associated inflammation in gastric organoids and a mouse model of *H. pylori* infection

**DOI:** 10.1007/s10787-026-02359-3

**Published:** 2026-08-10

**Authors:** Giulia Martinelli, Mariela Artola-Borán, Marco Fumagalli, Nicole Maranta, Giovanna Nicotra, Emma De Fabiani, Stefano Piazza, Enrico Sangiovanni, Mario Dell’Agli, Anne Müller

**Affiliations:** 1 Department of Pharmacological and Biomolecular Sciences (DiSFeB), “Rodolfo Paoletti”, University of Milan, Via Balzaretti 9, 20133, 02 50318403 Milan, Italy; 2 Institute of Molecular Cancer Research (IMCR), University of Zurich UZH, Strickhofstrasse 40a, CH-8057 Zurich, Switzerland; 3 Estratti Piante Officinali (EPO) S.R.L., Via Stadera 19, 20141 Milan, Italy

**Keywords:** *Castanea sativa* Mill., Castalagin, Ellagitannin, Food supplements, Gastritis, *Helicobacter pylori*

## Abstract

**Background:**

*Helicobacter pylori* is a bacterium that has been identified as a causative agent in the development of chronic gastritis, peptic ulcers, and gastric cancer. Current eradication therapies, based on antibiotic combination, are suffering from lack of patient compliance and primary antibiotic resistance. The traditional use and the scientific literature both report the gastroprotective role of natural extracts containing ellagitannins, suggesting an intriguing hypothesis about the potential impact on *H. pylori-*related gastritis. Anti-inflammatory and antibacterial effects of *Castanea sativa* Mill. extract standardized to contain castalagin have been previously demonstrated in human gastric epithelial cells.

**Aim:**

This experimental work aimed at investigating the role of a food grade chestnut leaf extract in complex models of *H. pylori* infection.

**Methods:**

The biological properties of chestnut leaf extract and castalagin have been validated, firstly in a novel murine gastric organoid cell model of *H. pylori* infection, and, secondly, in an in vivo infection model.

**Results:**

Some NF-κB pathway genes, including ligands for CXCR2 and CXCR3 receptors, were found to be suppressed by both the extract and the pure compound. Furthermore, both strongly suppressed *H. pylori* colonisation and gastric inflammatory mediators in mice. The infiltration of immune cells, mainly neutrophils and monocytes, was also reduced.

**Conclusion:**

The results of this work demonstrate the significant potential for the translation of innovative food supplements as co-adjuvants in the prevention and/or treatment of *H. pylori*-induced gastritis.

**Supplementary Information:**

The online version contains supplementary material available at https://doi.org/10.1007/s10787-026-02359-3.

## Introduction

*Helicobacter pylori* is a bacterium that has evolved sophisticated mechanisms to evade the bactericidal effects of gastric secretions, thus enabling its successful colonization of, and persistence in the human gastric mucosa. A variety of diseases associated with *H. pylori* infection, ranging from chronic gastritis to ulceration and cancer, can be ascribed to multifaceted interactions among bacterial virulence, host genetics, and environmental factors. Gastric cancer is the third largest cause of cancer death worldwide and the fifth most prevalent form of cancer. The lifetime risk of developing gastric cancer for individuals infected with *H. pylori* ranges from 1 to 5%, and approximately 90% of gastric cancers are associated with a past infection with *H. pylori*. Considering these factors, the World Health Organization has classified *H. pylori* as a type I carcinogen (Liou et al. 2020; Yamaoka 2010). The ability of the bacterium to colonise the stomach and to induce a series of inflammatory events, is due to a combination of virulence factors. The most relevant is the expression of the Cag pathogenicity island (*cag*PAI), transfers a protein effector, CagA, into the host cells (Naumann et al. 2017). The translocation of CagA has been shown to be responsible for the activation of the transcription factor NF-κB via the ALPK1-TIFA pathway. NF-κB in turn is involved in the regulation of various inflammatory genes and the activation of immune cells at the level of the gastric epithelium (Zimmermann et al. 2017). IL-8 is one of the crucial cytokines produced in this context, a powerful attractant for neutrophils, monocytes, macrophages, and dendritic cells into the gastric mucosa, and an active component in chronic-active gastritis. Furthermore, the IL-8/CXCR2 pathway has been demonstrated to play a pivotal role in the survival, invasion, and proliferation of cancer cells (Lee et al. 2012; Wilson and Crabtree 2007).

Antibiotics and acid-suppressive medications are used as an optimal clinical strategy to eradicate *H. pylori* infection. Nevertheless, the increasing antibiotic resistance and side effects contribute to therapy failure (Yamaoka 2024; Liu et al. 2023). For this reason, the primary objective of the present study was to identify novel *H. pylori* eradication approaches with an improved risk–benefit balance by investigating natural compounds with interest in complementary dietary applications. Several natural extracts from edible plants have shown in vitro antibacterial and anti-inflammatory activity at the gastric level during *H. pylori* infection. For instance, ellagitannin-rich extracts from strawberry and chestnut leaf have been shown to inhibit IL-8 and IL-6 release in *H. pylori*-infected GES-1 cells, with a partially NF-κB dependent mechanism of action, and to exhibit antibacterial activity against *H. pylori* (Martinelli et al. 2025; Piazza et al. 2023). Other ellagitannins display similar antibacterial effects (Funatogawa et al. 2004). The ethanolic extracts of chestnut leaf show antioxidant and antimicrobial properties, e.g. in the oxidative stress against UV-mediated DNA damage in keratinocytes (Cerulli et al. 2018), and antibacterial effect against methicillin-resistant *Staphylococcus aureus* (Salam et al. 2021). Its anti-inflammatory and anti-biofilm effect against *C. acnes* was attributed to the presence of the ellagitannin castalagin (Piazza et al. 2024). Despite the increased interest in the investigation of natural compounds in gastric inflammation, in vivo studies in *H. pylori*-infected models remain limited. A study has been conducted in an ethanol-induced gastric ulcer model in mice to demonstrate the gastroprotective efficacy of two *Quercus* leaf extracts and the pure ellagitannin castalagin (Khennouf et al. 2003). In a similar model employing rats, two ellagitannin-enriched extracts from *Rubus* showed gastroprotective activity, thereby supporting the hypothesis that ellagitannins might be included in dietary plans to prevent or treat gastritis (Sangiovanni et al. 2013).

Therefore, building on the previous study conducted in *H. pylori*-infected GES-1 cells (Piazza et al. 2023), we aimed to examine the potential activity of *Castanea sativa* Mill. (*C. sativa*) leaf and the ellagitannin castalagin in more complex models of *H. pylori* infection. Leaf from *C. sativa* has been recorded by several regulatory agency as botanical ingredient with nutritional and pharmaceutical value (e.g. FDA record IV3S2HH53G). This extract is a food grade extract rich in dietary polyphenols, especially tannins and flavonoids, and it is standardised to contain 1% castalagin. Infections of murine organoid cells and mice were carried out using a *cag*PAI-positive strain of *H. pylori.*

## Results

### Anti-*H. pylori* growth activity of *C. sativa* leaf extract and castalagin

**Fig. 1:**
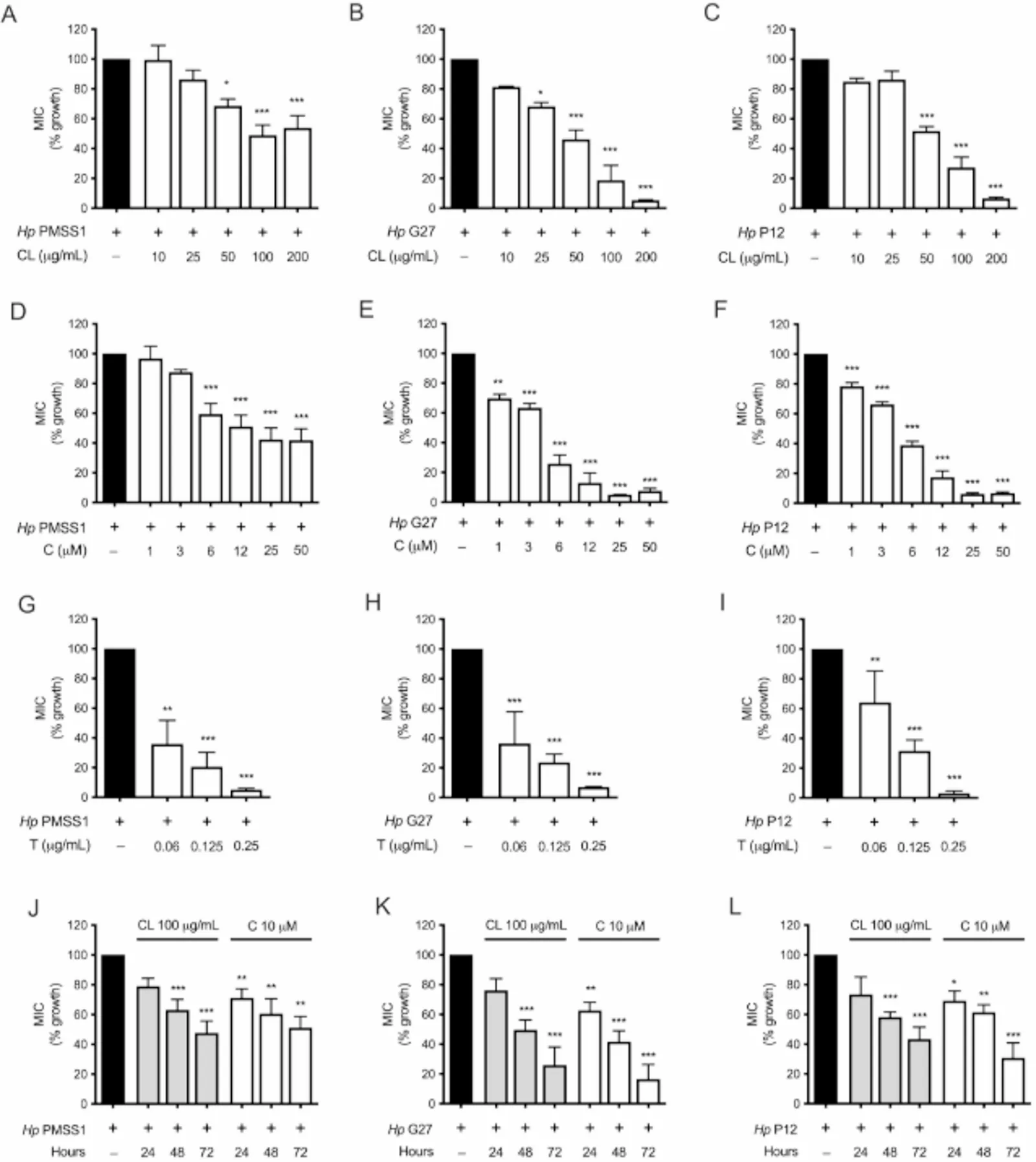
The anti-*H. pylori* growth activity of *C. sativa* leaf extract and castalagin. *H. pylori* PMSS1, G27, and P12 strains were cultivated in the presence of extract (CL) or pure molecule (C). Firstly, the anti-*H. pylori* growth activity of the extract (**A, B, C**) and pure molecule (**D, E, F**) at different concentration was measured by microbroth dilution assay after 72 h. Tetracycline (T) was utilised as the reference antibiotic (**G, H, I**), exhibiting a MIC of 0.06 μg/mL. Secondly, the anti-*H. pylori* growth activity of the extract (CL, 100 μg/mL, grey bars) and pure molecule (C, 10 μM, white bars) was measured by microbroth dilution assay over time, for 24, 48, or 72 h (**J, K, L**). The results are presented as the mean ± SEM (*n* = 4) and expressed as the relative percentage compared to *H. pylori* growth (black bars), which was arbitrarily assigned the value of 100%. The one-way ANOVA statistical test was employed, followed by the Bonferroni post-hoc test. * p < 0.05, **p < 0.01, and ***p < 0.001 vs. *H. pylori*

The anti-*H. pylori* growth activity of *C. sativa* leaf extract and castalagin was evaluated on the *cag*PAI-positive *H. pylori* strains PMSS1, G27, and P12. The differences of the three *H. pylori* strains in terms of virulence factors and susceptibility/resistance to antibiotics are shown in Table S1. The results were particularly promising for both the extract (Fig. 1A, B, C) and the pure molecule (Fig. 1D, E, F), showing a relevant inhibitory activity on bacterial growth at value of 50 μg/mL and 6 μM, respectively, for all *H. pylori* strains after 72 h of growth. Interestingly, this antibacterial activity was found to be comparable across the three strains examined, and consistent with previous results obtained with *H. pylori* strain 26695 (Piazza et al. 2023). Tetracycline, selected as a reference antibiotic, completely inhibited the bacterial growth at 0.25 μg/mL or 0.56 μM (Fig. 1G, H, I).

To gain a better understanding of the timing required for the action and efficacy of the extract and the pure molecule, the anti-*H. pylori* growth activity was additionally assessed over the time at the selected concentration of 100 μg/mL for chestnut leaf extract and 10 μM for castalagin (Fig. 1J, K, L). The time course experiment demonstrated that the antibacterial activity was already present at 24 h, although this effect was not statistically significant for the extract. The effect was significant at 48 h, reaching a maximum *H. pylori* growth inhibition at 72 h. Again, the activity observed among the three distinct *H. pylori* strains was comparable.

### In vitro anti-*H. pylori* colonisation and anti-inflammatory activity of *C. sativa* leaf extract and castalagin

The mechanisms of pathogenicity of *H. pylori* and related gastric inflammation have been widely studied in gastric epithelial cell lines, including the human GES-1 cells, by our research group (Martinelli et al. 2023a). Despite the numerous advantages offered by cell lines, they are not entirely representative of the complex environment of *H. pylori* infection. Gastric organoids are considered to be the in vitro model that most closely resembles the physiology of complete organs, mimicking the cellular diversity and the architectural complexity of the stomach (Idowu et al. 2022; Seidlitz et al. 2021).

The *cag*PAI-positive PMSS1 *H. pylori* strain was selected for organoid cell infection (MOI of 50). The chestnut leaf extract (concentration range: 50–200 μg/mL) and castalagin (concentration range: 25–100 μM) did not exhibit signs of cytotoxicity towards organoid cells after 72 h of treatment, as determined by the Trypan blue assay (Fig. S1).

**Fig. 2:**
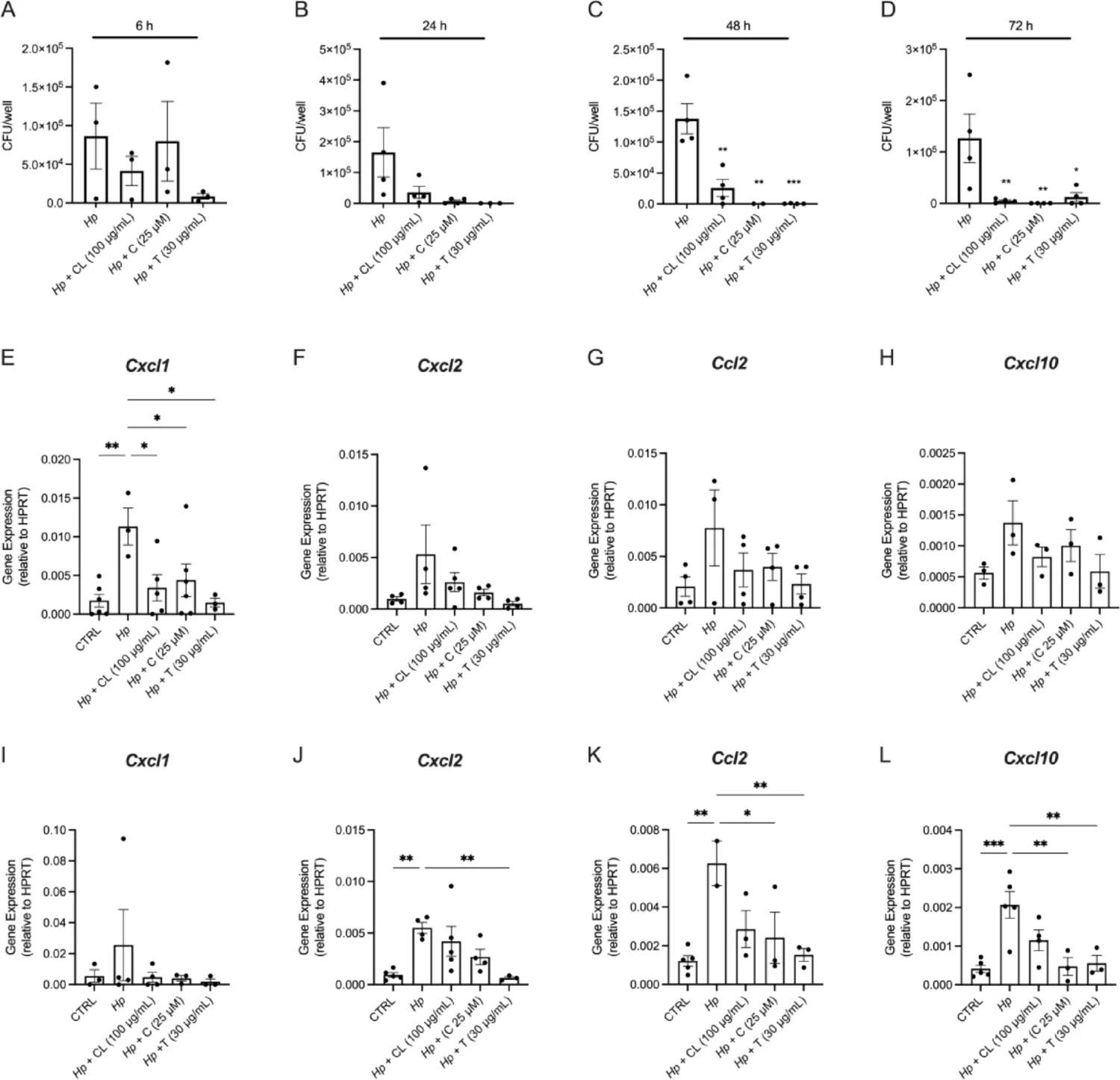
In vitro anti-*H. pylori* colonisation and anti-inflammatory activity of *C. sativa* leaf extract and castalagin on *H. pylori* PMSS1 cultured on murine gastric organoid cells. Firstly, 2D-organoid-derived monolayers were infected with *H. pylori* (*Hp*) for a period of 6 h and then were treated with the extract (CL, 100 μg/mL) or pure molecule (C, 25 μM) for a period of 6 (**A**), 24 (**B**), 48 (**C**), or 72 (**D**) hours. Thereafter, cells were harvested and plated into horse blood petri dishes. Following a period of 4 days, the formation of *H. pylori* colonies on the petri dishes was meticulously enumerated. The results are presented as the mean ± SEM of the absolute number of colonies counted per well following treatment with the extract or the pure molecule in a minimum of three experiments. Secondly, 2D-organoid-derived monolayers were infected with *H. pylori* for a period of 6 h and then were treated with the extract or pure molecule for a period of 24 (**E, F, G, H**) or 48 (**I, J, K, L**) hours. The gene expression on gastric tissue was measured with qPCR using the TaqMan method. The results were indicated as the mean ± SEM of the relative gene expression, calculated using the 2ΔC(t) method in a minimum of three experiments. Gene expression levels for each sample were normalized to HPRT expression. The one-way ANOVA statistical test was employed, followed by the Dunnett test. Tetracycline (T, 30 μg/mL) was utilised as the reference antibiotic. CTRL, untreated tissues, *p < 0.05, **p < 0.01, ***p < 0.001 vs. *H. pylori*

Following a 6-h exposure of 2D-seeded organoid cells to *H. pylori*, the extract (100 μg/mL) and the pure molecule (25 μM) were added to the co-culture for a further 6, 24, 48, or 72 h. From 24 h onward, both the extract and the pure molecule inhibited *H. pylori* viability in this model (Fig. 2A, B). This inhibition was clearly evident and statistically significant at 48 h (Fig. 2C) and at 72 h of treatment (Fig. 2D).

Since inhibition of IL-8 secretion and NF-κB translocation in *H. pylori*-infected GES-1 cells was previously demonstrated (Piazza et al. 2023), the inhibition of inflammatory genes responsible for leukocytes recruitment, particularly those linked to the NF-κB pathway and involved in CXCR2 and CXCR3 activation was evaluated. The expression of inflammatory genes in organoids, including *Cxcl1*, *Cxcl2*, *Cxcl10*, and *Ccl2*, has recently been evaluated in murine gastric organoids following stimulation of the NF-κB pathway with TNFα or ADP heptose (Wizenty et al. 2022), while it has never been evaluated in the *H. pylori* infected-WT murine gastric organoids.

Co-cultures of WT murine gastric organoids and *H. pylori* PMSS1, were treated with the extract (100 μg/mL) and the pure molecule (25 μM) for 24 h (Fig. 2E, F, G, H) or 48 h (Fig. 2I, J, K, L), followed by qRT-PCR. The results demonstrated that *Cxcl1*, the functional murine homologue of human *CXCL8* gene, was significantly inhibited at 24 h in respect to infected tissues (Fig. 2E), while *Cxcl2*, another functional murine homologue of human *CXCL8* gene, was statistically inhibited at the later time points (48 h) (Fig. 2J). Inhibitory effects were further observed on the expression of *Ccl2* and *Cxcl10* genes, which was more pronounced and significant after 48 h of treatment with castalagin (Fig. 2K, L).

*Cxcl11* and *Il6* were not expressed at 24 h, while they were slightly expressed and inhibited by the extract and pure molecule at 48 h (Fig. S2); the *Cxcl9* gene was not expressed at any time point considered. The expression levels of the genes under investigation were also evaluated after 72 h of treatment. The results showed that the *Cxcl1*, *Cxcl2*, *Ccl2,* and *Cxcl10* were expressed by *H. pylori*-infected gastric organoids, slightly reduced 72 h of treatment (Fig. S3). In contrast, the *Cxcl11*, *Il6,* and *Cxcl9* genes were not expressed.

These findings from our in vitro model of *H. pylori*-infected gastric organoids corroborated those previously obtained in *H. pylori*-infected GES-1 cells, thus confirming the antibacterial and the anti-inflammatory activities of *C. sativa* leaf extract and castalagin.

### In vivo anti-*H. pylori* colonisation and anti-inflammatory activity of *C. sativa* leaf extract and castalagin

To study potential antibacterial and anti-inflammatory activities of chestnut leaf extract and castalagin in an in vivo murine model, C57BL/6 WT mice were infected with the *H. pylori* PMSS1 strain for a period of 5 weeks. This period is necessary for the development of stable bacterial colonisation, which induces gastritis and triggers both local and systemic immune responses (Arnold et al. 2011; Artola-Borán et al. 2025). Thereafter, the mice were treated for a period of 10 days with the chestnut leaf extract (100 or 500 mg/kg) or castalagin (25 mg/kg). Gastric tissue of infected mice was used to assess *H. pylori* colonisation, the production of inflammatory mediators, and immune cell infiltration.

**Fig. 3:**
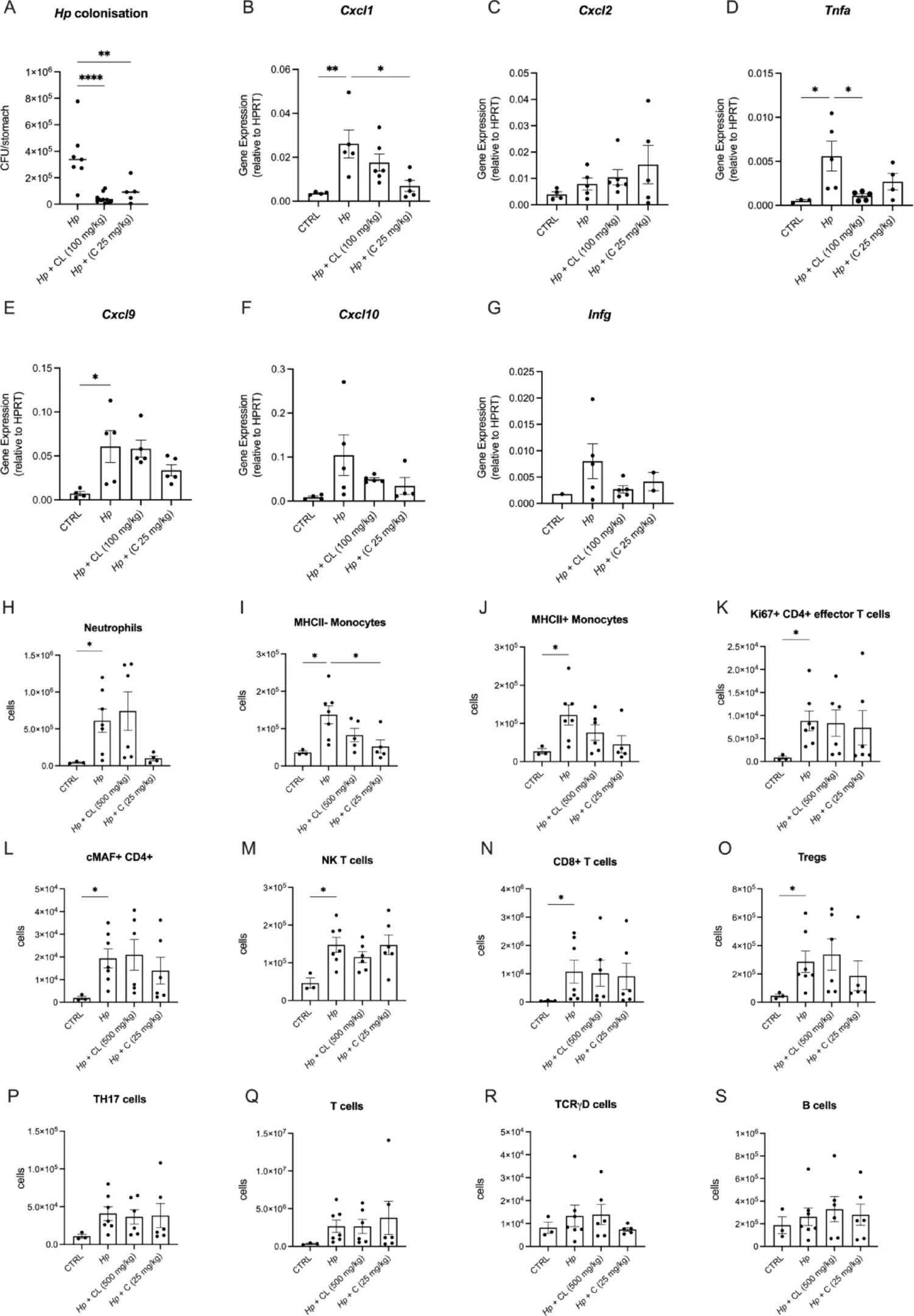
In vivo anti-*H. pylori* colonisation and anti-inflammatory activity of *C. sativa* leaf extract and castalagin in infected mice. WT mice were infected with *H. pylori* PMSS1 strain (*Hp*) for a period of 5 weeks and then were treated with the extract (CL, 100 mg/kg and 500 mg/kg) or pure molecule (C, 25 mg/kg) for the last 10 days of the infection. Thereafter, the stomachs were harvested, homogenized, and plated onto horse blood petri dishes for the evaluation of anti-*H. pylori* colonisation. *H. pylori* were enumerated 7 days later. Horizontal bars indicate medians. The non-parametric one-way ANOVA statistical test (Kruskal–Wallis) was employed, followed by the Dunn’s post-hoc test (**A**)*.* The inhibition of inflammatory gene expression was measured on gastric tissue with qPCR using the TaqMan method. The results are presented as the mean ± SEM of the relative gene expression, calculated using the 2ΔC(t) method. Gene expression levels for each sample were normalized to HPRT expression. One-way ANOVA statistical test was employed, followed by the Dunnett test (**B, C, D, E, F, G**). The inhibitory activity of the extract and castalagin was measured on neutrophil, monocyte, and lymphocyte infiltration into gastric tissue. The quantification of absolute counts per stomach by spectral flow cytometry is shown. The samples were then acquired on a 5-Laser Cytek© Aurora. The Kruskal-Wallis non-parametric test was employed, followed by the Dunn’s multiple comparisons test (H, I, J, K, L, M, N, O, P, Q, R, S). *p < 0.05, **p < 0.01, *** p < 0.001 vs. *H. pylori*

The extract (100 mg/kg) and the pure molecule exhibited a marked and significant inhibition of bacterial colonisation (Fig. 3A), corroborating the previously observed antibacterial activity.

*Cxcl1* was inhibited following treatment, with statistical significance for castalagin (Fig. 3B). Interestingly, *Tnfa, Cxcl9*, *Cxcl10,* and *Ifng* were also reduced, albeit nor significantly (Fig. 3D, E, F, G,). Other genes such as *Ccl2*, *Il1b*, *Il6*, and *Il17A*, were poorly expressed in the gastric mucosa of mice following 5 weeks of *H. pylori* infection.

To ascertain if the chestnut leaf extract and castalagin may have exerted a direct effect on the transcription factor NF-κB, two NF-κB inhibitor genes were also investigated. The expression of *Nfkbia* was unaffected by the treatments; however, the extract exhibited a slight inhibition of *Nfkbiz* gene expression, as an indirect consequence of NF-κB pathway inhibition (Fig. S4).

The immune response to infection, particularly the early recruitment of monocytes, neutrophils, macrophages, and dendritic cells to the site of inflammation, is directed by epithelial activation of NF-κB target genes. To study the most relevant myeloid, lymphocytic, T-cell, and granulocytic populations, we performed a flow cytometric analysis on single cell suspensions from gastric tissue for a comparison with colonisation data shown in Fig. 3A and Fig. S5. Mice were treated with chestnut leaf extract (500 mg/kg) or castalagin (25 mg/kg). As expected, the initial response to *H. pylori* was characterized by the presence of neutrophils and monocytes, which was effectively prevented by castalagin, while with a less extent by the chestnut leaf extract (Fig. 3H, I, J). Lymphocyte populations (e.g., Ki67 CD4 + , cMAF CD4 + , NKT, CD8 + , Tregs, Th17) were not affected by treatment (Fig. 3K, L, M, N, O, P, and Fig. S6).

## Discussion

In 2017, the WHO raised the attention on *H. pylori* as a high-priority infection for antibiotic research due to increasing antibiotic-resistance. The association between clarithromycin resistance and eradication failure has been documented (Savoldi et al. 2018). Clarithromycin and levofloxacin are still receiving high attention, since resistance is currently exceeding the 15% in 24 on 31 and 18 on 31 countries, respectively. Despite bismuth-based quadruple therapy is a solution to overcomes the problem, the access to drugs and screening tools remains a challenge for many countries still considering clarithromycin-based therapy as a first-line option (Schulz et al. 2025).

Especially for those countries, the search for botanicals with antimicrobial activities may represent a local answer (Liu et al. 2023). Polyphenols including ellagitannins from diverse natural matrices with nutritional value, such as red berries, pomegranate, and chestnut tree, were reported for anti-*H. pylori* and anti-inflammatory activity (Martinelli et al. 2025; Sangiovanni et al. 2013; Piazza et al. 2023; Funatogawa et al. 2004; Mayyas et al. 2021). However, the paucity of experimental evidence in complex models of *H. pylori* infection limits the translation to clinics.

Our previous studies showed that chestnut leaf extract and its ellagitannins, castalagin and vescalagin, inhibit *H. pylori*-induced inflammation in human gastric epithelial cells (GES-1) and possess antibacterial and anti-adhesive properties. In the present work, chestnut leaf extract and castalagin inhibited the growth of three different cagPAI positive *H. pylori* strains, known for different antibiotic susceptibility. Accordingly, the extract (100 μg/mL) and the pure compound (25 μM) were effective in suppressing the colonisation of murine organoid cells by *H. pylori*. They showed anti-inflammatory activity, with inhibitory mechanisms related to NF-κB target genes, having roles in neutrophil and monocyte recruitment, such as *Cxcl1* (the functional mouse homologue of *CXCL8*), *Ccl2*, and (Hughes and Nibbs 2018). The antibacterial and anti-inflammatory properties of chestnut leaf extract (100 mg/kg or 500 mg/kg) and castalagin (25 mg/kg) were also validated in an in vivo study using *H. pylori*-infected C57BL/6 WT mice. Again, the results demonstrated a significant activity in inhibiting the colonisation of *H. pylori* in the complex organism. Furthermore, a decrease of NF-κB-dependent inflammatory mediators was suggested, particularly in the treatment with castalagin, including *Cxcl1, Tnfa*, *Cxcl10*, *Cxcl9*, and *Ifng*. The inhibition of NF-κB target genes has also been linked to an impaired immune response to infection. Therefore, a thorough investigation of the most relevant immune cells was conducted, which revealed that castalagin diminished the number of monocytes and neutrophils in the gastric mucosa. These populations are implicated in the primary response to *H. pylori*; thus, the reduction of infiltration plausibly reflected the combination of antibacterial and anti-inflammatory effect at mucosal level*.* However, an impaired colonisation was also observed in organoids, thus suggesting *H. pylori* as the main target of our candidates.

It is noteworthy that in vitro effective concentrations were translated into in vivo effective doses: the underlying reason, regardless of the reliability of models, plausibly resides in the stability of ellagitannins in the gastric environment, in line by previous works (Piazza et al. 2023; Martinelli et al. 2025). We thus propose chestnut leaf extract or its active compound as a botanical adjuvant with potentially low risk of resistance, aimed at supporting the eradication therapy. However, specific studies are demanded to identify the ideal form of administration, including food supplementation and pharmaceutical formulation. Moreover, future research will be required to characterize the antibiotic mechanisms and address the relevance of doses for humans. To the best of our knowledge this is the first study evaluating the potential properties of a chestnut leaf extract and castalagin in relation to *H. pylori *in vivo infection, making these results highly promising for dietary intervention and clinical investigation.

## Materials and methods

### *Castanea sativa* Mill. leaf extract

*C. sativa* leaf extract was provided by EPO S.r.l. (Milan, Italy). The chestnut leaf extract was an hydroethanolic (EtOH:H2O 25:75) extract (Martinelli et al. 2023b). The ellagitannin castalagin (purity > 95%, Phytolab GmbH & Co. KG, Vestenbergsgreuth, Germany) was detected in the methanol solution of chestnut leaves extract and further quantified by UPLC–MS analysis in a previous publication (approximately 1% *w/w* of the dry extract) (Piazza et al. 2023).

### *H. pylori* cultures

*H. pylori cag*PAI + strains PMSS1 (Arnold et al. 2011), G27 (Xiang et al. 1995), and P12 were grown on Columbia horse blood agar plates for two days and then in liquid culture using Brucella broth supplemented with 10% FBS and vancomycin 10 μg/mL for three days. All the bacteria were incubated in a microaerophilic atmosphere (5% O2, 10% CO2, and 85% N2) at 37 °C with 100% humidity. The cultures were frequently evaluated by light microscopy for contamination, morphology, and motility. An OD value of 0.1, measured at 600 nm using a spectrophotometer, is equivalent to 5 × 10^7^ bacteria. All bacterial strains were maintained in 70% Brucella broth and 30% glycerol at -80 °C.

### Organoids culture and treatments

The organoid culture protocol utilized in this study was previously published (He et al. 2023). In brief, the glands of C57BL/6N WT mice were collected from the antrum of the stomach, placed in DMEM medium, and seeded into 24-well plates with Matrigel solution. The 3D organoids were then cultivated at 37 °C in ADMEM medium, which was supplemented with 10 mM penicillin/streptomycin, Wnt-conditioned medium (50%) from Wnt-3A cell culture (ATCC©, CRL-2647™), R-spondin-1 (1 μg/mL), N-acetylcysteine (1 mM), EGF (50 ng/mL), FGF (100 ng/mL), Noggin (100 ng/mL), Gastrin (10 nM), N2, B27, HEPES (10 mM), Y27632 (10 μM), and GlutaMAX (2 mM). For 2D seeding, the 3D murine gastric organoids were dissociated, and the single-cell suspension was seeded into a 24-well plate (1.5 × 10^4^ cells/well) or a 6-well plate (7.5 × 10^4^ cells/well), prior coated with rat tail collagen. The 2D medium is identical to the 3D medium, with the exception of the omission of the Wnt-conditioned medium, R-spondin, penicillin/streptomycin, FGF, Noggin, and N-acetylcysteine, and supplemented with 10% FBS and 1 μM TGFβ inhibitor. The organoid cells were infected with *H. pylori* for 6 h at MOI of 50. Subsequently, chestnut leaf extract and castalagin were added at concentrations of 100 μg/mL and 25 μM, respectively, and their anti-inflammatory and antibacterial properties were then studied after 6–24-48–72 h. The vehicle (DMSO) was normalized in the untreated conditions. Tetracycline (30 μg/mL, 67.50 μM) was used as a reference antibiotic.

### Animal experimentation

C57BL/6 WT female mice were procured from Janvier at 6–7 weeks of age, bred, and maintained under specific pathogen-free conditions in accredited animal facilities at the University of Zürich. The mice were arranged randomly to groups of five per cages, with *ad libitum* access to water and food. The mice were infected orally via gavage on two consecutive days with 10^8^ CFU of the *H. pylori cag*PAI + strain PMSS1. During the last 10 days of a 5-week period of infection, the mice were treated daily with 100 mg/kg or 500 mg/kg of chestnut leaf extract and 25 mg/kg of castalagin. Mice were monitored twice per week. As expected, no negative effects of the infection on overall health of the animals were detected. The stomachs of the animals were collected at the end of the study, and gastric tissue was evaluated for the *H. pylori* colonization, the expression of inflammatory genes, and the infiltration by immune cells. All animal experimentation adhered to federal, cantonal and institutional guidelines and was reviewed and approved by the Zurich Cantonal Veterinary office (ZH023/2025).

### Antibacterial activity

#### Minimum inhibitory concentration (MIC)

A microbroth dilution method was used to test the antibacterial activity of chestnut leaf extract and castalagin at varying concentrations (ranging from 10–200 μg/mL for extract, and 1–50 μM for castalagin), using the method previously described in (Piazza et al. 2023). A quantity of *H. pylori* suspension (OD = 0.1) was added to each 96-well U-bottom plate and the plate was incubated at 37 °C under conditions of microaerophilicity for 24–48-72 h. The bacterial growth rate was subsequently measured at 600 nm, using a spectrophotometer. Tetracycline (MIC = 0.125 μg/mL) was used as a reference antibiotic.

#### *H. pylori* colonisation

The colonization of *H. pylori* after treatment with chestnut leaf extract and castalagin was evaluated in gastric organoid cells and in mice. Subsequent to the designated treatment period, the cells were harvested in PBS 1 × , while the stomachs of the mice were homogenised in Brucella broth using a tissue homogenizer. A volume of 2 μL or 10 μL of the solution was plated onto horse blood agar plates, which were supplemented with the following antibiotics and antifungals: vancomycin (10 μg/mL), cefsulodin (5 μg/mL), polymyxin B sulphate (2.5 U/mL), trimethoprim (5 μg/mL), amphotericin B (8 μg/mL), nalidixic acid (15 μg/mL), bacitracin (100 μg/mL), and β-cyclodextrin (200 μg/mL). The colonies formed were enumerated following a five-day culture period under microaerophilic conditions at 37 °C and the percentage of *H. pylori* survival post-treatment was calculated.

### Cytotoxicity (Trypan blue assay)

The organoid morphology and integrity were evaluated by light microscopy prior to and following each treatment, while cell viability in response to chestnut leaf extract and castalagin was quantified using the Trypan blue assay. In summary, cells were seeded in a 24-well plate for 48 h, and viability was measured after a 6-h treatment with extract or ellagitannin. The cells were harvested and diluted 1:2 in trypan blue solution (0.1 mg/mL, PBS 1 ×). After 1–2 min the live cells (white) and the dead cells (blue) were counted using a Neubauer haemocytometer.

### Gene expression

The expression levels of the genes *Cxcl1, Cxcl2, Ccl2, Tnfa, Il1b, Cxcl9, Cxcl10, Cxcl11, Ifng, Il6, Il17A, Nfkbiz, Nfkbia, RegIIIγ, and S100A9*, were evaluated in gastric organoid cells or animal gastric mucosa both infected with the *H. pylori cag*PAI + strain PMSS1 and treated with extract or castalagin. The RNA was extracted from the organoid cells using the miRNeasy Mini Kit (QIAGEN), while from gastric mucosa using the NucleoSpin™ RNA Mini (Machery-Nagel™) in accordance with the manufacturer’s instructions. A complementary DNA sample was prepared, quantitative PCR was conducted using the TaqMan™ method and analyzed with a Light Cycler 480 detection system (Roche).

### Leukocyte isolation and flow cytometer analysis

Following the in vivo mice infection with *H. pylori* and subsequent treatments with chestnut leaf extract (500 mg/kg) or castalagin (25 mg/kg), leukocytes were isolated from the lamina propria of the stomachs of mice, stained with antibodies, fixed, and permeabilized as described in (Artola-Borán et al. 2025). The samples were acquired on a 5-laser Cytek® Aurora (Cytek Biosciences) equipped with 64 detectors. The data were analysed using SpectroFlow v3.0 (Cytek Biosciences), FlowJo v.10 software (FlowJo/BD) and R (version 4.3.2, R Core Team, 2021) using the FlowCore Bioconductor R package (version 2.14.2.).

### Statistical analysis

The data were analysed using GraphPad Prism software (version 9.0), employing a one-way ANOVA test followed by a Bonferroni or Dunnett post-hoc analysis, or a non-parametric one-way ANOVA (Kruskal–Wallis) test followed by a Dunn’s post-hoc analysis, depending on the Gaussian distribution of the experimental data. The results were expressed as the mean ± SEM of at least three independent experiments for antibacterial assays and for gastric organoid cells, and as the median or mean ± SEM of all animals (excluding outliers) for the animal experiment. The following levels of significance were used: * p < 0.05, ** p < 0.01, and *** p < 0.001.

## Supplementary Information

Below is the link to the electronic supplementary material.

Supplementary Material 1

## Data Availability

No datasets were generated or analysed during the current study.

## Abbreviations

ALPK1-TIFA: Alpha protein Kinase 1-TRAF-interacting forkhead-associated protein A
C: Castalagin
*cag*PAI: Cag Pathogenicity Island
CCL-: CC class chemokine-
CL: Chestnut leaf extract
CXCL-: CXC class chemokine-
CXCR-: C-X-C motif chemokine receptor-
IFN-: Interferon-
IL-: Interleukin-
NF-κB: Nuclear factor kappa-light-chain-enhancer of activated B cells
NFKBI*-*: NFKB inhibitor
