# Supplementary material for: Chestnut (Castanea sativa Mill.) leaf extract and pure castalagin reduce bacterial colonisation and the associated inflammation in gastric organoids and a mouse model of H. pylori infection
Source: Inflammopharmacology. 2026 Aug 10;34(9):6265–74. doi: 10.1007/s10787-026-02359-3 (PMC13558433; doi:10.1007/s10787-026-02359-3)
Supplement: Supplementary file 1 [file 10787_2026_2359_MOESM1_ESM.docx]

**Supplementary**

1. The differences of the three *H. pylori* strains used in terms of virulence factors and susceptibility/resistance to antibiotics

| ***H. pylori* strain** | ***cag*PAI** | ***cagA*** | ***vacA*** | **Antibiotical susceptibility** |
| --- | --- | --- | --- | --- |
| PMSS1 | + | + | + | MTZ, T |
| G27 | + | + | + | T, AMX, CLR, MTZ, LVX |
| P12 | + | + | + | T |

**Table S1**. The differences of *cag*PAI positive PMSS1, G27, and P12 *H. pylori* strains in terms of virulence factors and susceptibility/resistance to antibiotics. N.S. = not studied; T: Tetracycline; LVX: Levofloxacin; CLR: Clarithromycin; MTZ: Metronidazole; AMX: Amoxicillin.

2. *In vitro* anti-inflammatory activity of *C. sativa* leaf extract and castalagin

**Figure S1**. 2D-organoid-derived monolayers viability following chestnut leaf extract (CL, concentration range 200-50 μg/mL, grey bars) and castalagin (C, concentration range 100-25 μM, white bars) treatment for a period of 72 hours. The cell viability was measured with Trypan blue assay. The results are presented as the mean ± SEM (*n*=3) and expressed as the relative percentage compared to CTRL, which was arbitrarily assigned the value of 100 %. The one-way ANOVA statistical test was employed, followed by the Bonferroni post-hoc test.

**Figure S2**. The inhibitory activity of *C. sativa* leaf extract and castalagin on *CXCL11* and *IL6* genes following *H. pylori*-infected organoid cells. 2D-organoid-derived monolayers were infected with *H. pylori* (*Hp*) for a period of 6 hours and then were treated with the extract (CL, 100 μg/mL) or pure molecule (C, 25 μM) for a period of 48 hours. The gene expression was measured with qPCR using the TaqMan method. The results were indicated as the mean ± SEM of the relative gene expression, calculated using the 2ΔC(t) method in a minimum of three experiments. Gene expression levels for each sample were normalized to HPRT expression. The one-way ANOVA statistical test was employed, followed by the Dunnett test. Tetracycline (T, 30 μg/mL) was utilised as the reference antibiotic. *p < 0.05, **p < 0.01 vs. *H. pylori*.

**Figure S3**. The inhibitory activity of *C. sativa* leaf extract and castalagin on *CXCL1, CXCL2, CCL2,* and *CXCL10* genes following *H. pylori*-infected organoid cells. 2D-organoid-derived monolayers were infected with *H. pylori* (*Hp*) for a period of 6 hours and then were treated with the extract (CL, 100 μg/mL) or pure molecule (C, 25 μM) for a period of 72 hours. The gene expression was measured with qPCR using the TaqMan method. The results were indicated as the mean ± SEM of the relative gene expression, calculated using the 2ΔC(t) method in a minimum of three experiments. Gene expression levels for each sample were normalized to HPRT expression. The one-way ANOVA statistical test was employed, followed by the Dunnett test. Tetracycline (T, 30 μg/mL) was utilised as the reference antibiotic.

3. *In vivo* anti-inflammatory activity of *C. sativa* leaf extract and castalagin

**Figure S4.** The activity of *C. sativa* leaf extract and castalagin on two NF-κB inhibitors following *H. pylori* infection in mice. Mice were infected with *H. pylori* (*Hp*) for a period of 5 weeks and then were treated with the extract (CL, 100 mg/kg) or pure molecule (C, 25 mg/kg) for a period of 10 days. *NFKBIA* is a gene encoding a member of the NF-κB inhibitor family that inhibits NFKB/REL complexes, while *NFKBIZ* gene is induced by LPS and is responsible for activating nuclear I kappa B and IL-6 production during *H. pylori* infection. The gene expression was measured with qPCR using the TaqMan method. The results were indicated as the mean ± SEM of the relative gene expression, calculated using the 2ΔC(t) method. Gene expression levels for each sample were normalized to HPRT expression. The one-way ANOVA statistical test was employed, followed by the Dunnett test.

**Figure S5.** The anti-*H. pylori* colonisation activity of *C. sativa* leaf extract in infected mice. WT mice were infected with *H. pylori* PMSS1 strain (*Hp*) for a period of 5 weeks and then were treated with the extract (CL, 500 mg/kg) for the last 10 days of the infection. Thereafter, the stomachs were harvested, homogenized, and plated onto horse blood petri dishes. *H. pylori* were enumerated 7 days later. Horizontal bars indicate medians. The non-parametric one-way ANOVA statistical test (Kruskal-Wallis) was employed, followed by the Dunn’s post-hoc test. **p < 0.01, ****p < 0.001 vs*. H. pylori.*

**Figure S6.** The inhibitory activity of *C. sativa* leaf extract and castalagin on various immune cells responses following *H. pylori* infection in mice. Mice were infected with *H. pylori* (*Hp*) for a period of 5 weeks and then were treated with the extract (CL, 500 mg/kg) or pure molecule (C, 25 mg/kg) for a period of 10 days. The quantification of the immune cells (in terms of absolute counts per stomach) was obtained by isolating leukocyte from the lamina propria and staining the single-cell suspension with various antibodies. The samples were then acquired on a 5-Laser Cytek© Aurora. The Kruskal-Wallis non-parametric test was employed, followed by the Dunn’s multiple comparisons test. * p < 0.05, ** p < 0.01 vs. *H. pylori.*
